# Grounding, necessity, and relevance

**DOI:** 10.1007/s11098-023-01968-w

**Published:** 2023-07-03

**Authors:** Salim Hirèche

**Affiliations:** 1 Université de Genève, Geneva, Switzerland; 2 Université de Neuchâtel, Neuchâtel, Switzerland

**Keywords:** Grounding, Necessity, Relevance, Explanation, Determination, Totality facts

## Abstract

*Grounding necessitarianism* (GN) is the view that full grounds necessitate what they ground. Although GN has been rather popular among philosophers, it faces important counterexamples: For instance, A = [Socrates died] fully grounds C = [Xanthippe became a widow]. However, A fails to necessitate C: A *could* have obtained together with B = [Socrates and Xanthippe were never married], without C obtaining. In many cases, the debate essentially reduces to whether A indeed *fully* grounds C–as the contingentist claims–or if instead C is fully grounded in A^+^, namely A *plus* some supplementary fact S (e.g. [Xanthippe was married to Socrates])–as the necessitarian claims. Both sides typically agree that A^+^ necessitates C, while A does not; they disagree on whether A or A^+^ fully grounds C. This paper offers a novel defence of the claim that, in these typical cases, unlike A^+^, A fails to fully ground C–thereby bringing further support to GN. First and foremost, unlike A^+^, A fails to fully ground C because it fails to contain just what is *relevant* to do so, in two distinct senses–*explanatory* and *generative* relevance. Second, going for A, rather than A^+^, as a full ground undermines not just grounding *necessitarianism*, but modally weaker views which even contingentists may want to preserve.

## Introduction

For the last two decades, the notion of metaphysical grounding has played an increasingly important role in metaphysics and across philosophy.^1^ Unsurprisingly, then, many recent debates have centred around the question of what grounding is, and what its core features are. One issue concerns its modal status: Do full grounds *necessitate* what they ground? The view that they do is known as *grounding necessitarianism* (GN): if A fully grounds C, then A necessitates C.^2^ While GN has been rather popular among grounding theorists, it is disputed, facing various alleged counterexamples.^3^ For instance (using “[p]” for “the fact that p”), A = [Socrates died] fully grounds C = [Xanthippe became a widow]. However, A fails to necessitate C: A *could* have obtained together with B = [Socrates and Xanthippe were never married], without C obtaining.

In many cases, the debate essentially reduces to whether A indeed *fully* grounds C—as the contingentist^4^ claims – or if A only partially grounds C, and C is instead fully grounded in A^+^, namely A *plus* some supplementary fact S (e.g. [Xanthippe was married to Socrates])—as the necessitarian claims. Both sides can, and typically do, agree that A at least partially grounds C; that A does not necessitate C, since the scenario where A obtains with B but without C is genuinely possible; and that A^+^ does necessitate C. They disagree on whether A or A^+^ fully grounds C. The contingentist typically claims that, at best, S is a “background condition”, or *enabler*—it enables A to fully ground C, without being itself part of the full ground. By contrast, the necessitarian claims that, just like A, S is part of the full ground, acting as a *completer*—it completes A to get a full ground for C.^5^

This paper offers a novel defence of the completer view—thereby bringing further support to GN. After setting the stage for the discussion (§2), I present three arguments (§3). Two are direct: the alleged non-necessitating full ground, A (unlike the completed one, A^+^) fails to contain just what is *relevant* to fully ground C, in two distinct senses—*explanatory* relevance (§3.1) and *generative* relevance (§3.2). My third argument is indirect: going for A (rather than A^+^) as a full ground undermines, not just grounding *necessitarianism*, but modally weaker views which even grounding contingentists may want to preserve (§3.3).

## Setting the stage

Throughout the discussion, I will refer to single facts with capital letters (“A”), and to (strict or non-strict) pluralities of facts with double capital letters (“AA”). I will assume that grounding is a relation which may hold between a plurality of facts AA (the grounding facts) and a single fact C (the grounded fact).^6^ And I will use the following common formulation of GN:

**Grounding Necessitarianism (GN)** For any plurality of facts AA and fact C, if AA fully grounds C, then ☐(AA → C),

where → is the material conditional, ☐ the metaphysical necessity operator (with standard possible worlds semantics), and “☐(AA→C)” is short for “☐(all AA obtain → C obtains)”.

In general, a counterexample to GN is a possible scenario where AA obtain and C does not—i.e. ◇(AA∧¬C). Typically, alleged counterexamples are slightly more complex, involving further facts BB obtaining with AA and “blocking” the obtaining of C—i.e. ◇(AA∧BB∧¬C).^7^ Both types of scenarios, if genuinely possible, clearly constitute counterexamples to GN if necessitation is understood in (something like) the above classical way. (The necessitarian might invoke a non-classical notion of necessitation to deny that such possible scenarios falsify GN, but I will not pursue this route.^8^)

I will focus on an important class of potential counterexamples. Here are some illustrations.

*Accidental generalization*: C=[∀x(Fx→Gx)] is fully grounded in AA=[Ga], [Gb], … – where *a*, *b*, … are the actual F’s. But there is a possible blocker: AA *could* have co-obtained with B=[¬Gc], for some possible *c* that would have been F. (See Skiles, 2015: §4)

For convenience, we will work with a concrete example:

*Squirrels*: C=[All mammals in Rovena’s garden are squirrels] is fully grounded in AA=[Tim is a squirrel], [Tom is a squirrel] – where Tim and Tom are the actual two mammals in her garden. But there could have been an additional, non-squirrel mammal in her garden: say, a hedgehog.

Let us move on to different cases:

*Taste*: My having a pleasant tasting experience – fact C – is fully grounded in my tasting chocolate^9^– fact A. Yet, I could have been tasting *both* chocolate and fish at once, in which case my tasting experience might not have been so pleasant – A would have co-obtained with B (my tasting fish), without C obtaining.

*Widow*: A=[Socrates died] fully grounds C=[Xanthippe became a widow], but there is a possible world where B=[Xanthippe and Socrates were never married] obtains and “blocks” C. (Trogdon, 2013a: 477-8; adapted from Schnieder, 2006b)

*Obligation*: A=[I promised to φ] fully grounds C=[I ought to φ], but there could have been a blocker: B_1_=[My promise was given under duress], or B_2_=[I’m not able to φ]. (Trogdon, 2013a: 477-8; adapted from Dancy, 2004, ch. 3)

Beyond such illustrations, the type of cases to be addressed may be characterized as those for which the following assumptions are plausible, and can be–indeed typically are–accepted by necessitarians and contingentists alike. First, the contingentist’s scenario is genuinely possible–i.e. ◇(AA∧BB∧¬C).^10^ For instance, it would seem hopeless for the necessitarian to insist that there could not genuinely have been an additional (non-squirrel) mammal in Rovena’s garden. Second, AA at least *partially* grounds C – e.g. it seems implausible that [Socrates died] may not even partially ground [Xanthippe became a widow]. Indeed, we may grant the contingentist that AA is a *prima facie* reasonable candidate *full* ground for C.

Third, there is a plurality SS which can plausibly be added to AA to get another *prima facie* reasonable candidate: AA^+^ = AA, SS. For *widow*, an obvious candidate is S = [Xanthippe was married to Socrates]; for *obligation*, the plurality SS = [I’m able to φ], [My promise to φ was not given under duress]; for *accidental generalization*, the totality fact^11^ S = [*a*, *b*,… are the only F’s]; for *taste*, S = [Chocolate is *all* I’m tasting]. Fourth, unlike AA, AA^+^ does necessitate C—at least necessitation is not threatened by the alleged blocker. In *widow*, AA^+^ = [Socrates died], [Xanthippe and Socrates were married] is incompatible with B = [Xanthippe and Socrates were never married]—so that we do *not* have ◇(AA^+^∧B∧¬C).

Fifth, for simplicity, we may further assume that AA and AA^+^ are the *only* (serious) candidate full grounds for C (any other candidate is either a non-necessitating variant of AA, or a necessitating variant of AA^+^). On these assumptions, the debate entirely reduces to whether AA fully grounds C: we have a genuine counterexample to GN if and only if it does. In particular, it is irrelevant whether AA^+^ fully grounds C: if it does not, we cannot conclude that AA does (C could have *no* full ground); if AA^+^ does fully ground C, we cannot conclude that AA does not (C could be fully grounded in *each*).

Yet, contingentists and necessitarians typically *also* make claims about AA^+^ = AA, SS. For the contingentist, AA^+^ does not fully ground C: SS, at best, serves as an *enabler*—helping AA fully ground C. For the necessitarian, by contrast, AA^+^ fully grounds C—SS being a *completer*. The reason why the status of AA^+^ is typically discussed, and why I will also discuss it here, is that it *does* become relevant under the following *further* (sixth and seventh) typical assumptions. On the one hand, C *is* fully grounded (e.g. it seems implausible that [Xanthippe became a widow] has *no* full ground).^12^ On the other hand, C is grounded in *at most* AA or AA^+^, *not* in each (the idea being that, since AA^+^ strictly includes AA, they cannot both be full grounds on pain of one containing more or less than just what is relevant^13^). Under these further assumptions, both sides may strengthen their own cases. Assuming that C *is* fully grounded in the first place, if AA^+^ does not fully ground C, then it should be AA. Thus, beyond arguing *directly* that AA fully grounds C, the contingentist can follow a second, indirect route–through arguing that AA^+^ does not. Likewise, assuming that C is *not* fully grounded in each of the two candidates, if AA^+^ fully grounds C, then AA does not. Thus, in addition to arguing *directly* that AA does not fully ground C, the necessitarian can follow another, indirect route–through arguing that AA^+^ does. Accordingly, in this paper, I will primarily argue that AA does not fully ground C, but also that AA^+^ does – i.e. to get a full ground for C, it is both necessary and sufficient to *complete* AA with SS.

This completer view has been invoked by necessitarians.^14^ Yet, it is disputed^15^—and in need of further argument. In providing a novel defence of it in the specific type of cases described above, I will offer a partial defence of GN (against one type of alleged counterexamples) rather than a fully general one (against *any* type of alleged counterexamples).^16^ However, arguably, the type considered here plays a particularly important role in the debate–covering a large part of the counterexamples found in the literature. Moreover, the completer strategy pursued here is widely applicable. For one thing, counterexamples to GN almost always involve some further BB “interfering” with AA fully grounding C, and in principle one can find some SS incompatible with BB—no one seems to have come up with a case for which there would be *no* reasonable completer.^17^ Thus, although I will focus on a type of cases to which my arguments apply most directly, they may apply or adapt to different ones.^18^ For those reasons, though specific and thereby inevitably partial, my proposed defence, if convincing, will bring significant support to GN.

## Arguing for the completer view

### Explanatory relevance: verbal explanations and implicit content

Grounding–unlike e.g. mere (strict) implication–is a relation whose relata meet certain *relevance* conditions.^19^ More precisely, I will rely on the idea that a full ground should contain *exactly* what is relevant for it to ground what it grounds–i.e. no more and no less than what it needs to do so. This principle sounds plausible, and it is acceptable to contingentists and necessitarians alike. Indeed, typically, the contingentist will claim that AA meets the condition, while AA^+^ contains *too much* to contain *only* what is relevant; the necessitarian will claim that AA^+^ meets the condition, while AA contains *too little* to contain *everything* relevant.

Instead of pursuing this line of argument as such, I will break it into two parts, focusing first on *explanatory* relevance, then on *generative* relevance (I use terms such as “generation”, “bringing about”, “building” or “determination” interchangeably). Relying on more specific relevance principles will make the discussion more precise. Moreover, the two arguments may convince different readers. In particular, there are two main positions on the relation between grounding and explanation: for “unionists”,^20^ grounding is (primarily) a form of explanation (“explanation_G_”); for “separatists”,^21^ grounding is (primarily) a form of generation (“generation_G_”).^22^ Separatists may be more easily convinced by my argument concerning generative relevance (§3.2); and unionists, by my argument concerning explanatory relevance, to which I now turn.

It is widely thought that grounds provide mind-independent, metaphysical explanations for what they ground – either by just *being* such explanations^23^ or by “backing” them.^24^,^25^ It seems plausible, more specifically, that a ground contains *only* facts that are relevant to explain what it grounds,^26^ and that a *full* ground, furthermore, contains *all* the facts relevant to fully explain what it grounds.^27^ In other words, a full ground contains *just* what is relevant for it to fully explain what it grounds – no more and no less than what it needs to do so. This suggests an explanatory version of the abovementioned general relevance principle:

**(GROUND-EXPLANATION)** If AA fully grounds C, then AA contains just what is relevant to fully explain C.

(GROUND-EXPLANATION) is very likely to be accepted by unionists—if grounding just *is* explanation_G_, then it is difficult to see how, for instance, AA may fully ground C *without* fully explaning_G_ C. As regards separatists, some may dispute the principle^28^ (they may, however, be more convinced by my argument in §3.2, which relies on an analogous principle linking grounding with *generation*). Importantly, (GROUND-EXPLANATION) is acceptable to grounding contingentists and necessitarians alike. The former will typically argue that AA (the alleged non-necessitating full ground) meets the condition, while AA^+^ contains *too much*; the latter, that AA^+^ meets the condition, while AA contains *too little*.

A common way to assess whether AA, a candidate ground full ground for C, satisfies a condition like (GROUND-EXPLANATION) is to rely on our intuitions concerning a corresponding *verbal* explanatory proposal for C citing the facts in AA (see Leuenberger, 2014a: 151–2; Wilson, 2018: 728–9), namely a proposal of the form “*c* because *a*_*1*_*, a*_*2*_*, …*” (where C = [c] and AA = [a_1_], [a_2_], …)—call it the “AA-proposal”. The criterion may be expressed as follows:

(**Explanation-Proposal**) AA contains just what is relevant to fully explain C *iff* one would accept the AA-proposal as containing just enough for a fully satisfactory answer to the question of why C obtains.

I will argue that criteria like (Explanation-Proposal) are importantly flawed—making the contingentist’s preferred candidate full ground look more plausible than it really is –, and defend an alternative criterion—on which the apparent plausibility disappears. But it will be instructive to first see (Explanation-Proposal) at work.

For *taste*, the A-proposal is “I’m having a pleasant tasting experience because I’m tasting chocolate”, which may indeed sound like containing just enough for a fully satisfactory answer. Accordingly, in the AA^+^-proposal, “I’m having a pleasant tasting experience because I’m tasting *just* chocolate (or: chocolate *and nothing else)*”, the addition may sound superfluous. For *squirrels*, the AA-proposal, “All mammals in Rovena’s garden are squirrels because are Tim and Tom are squirrels”, where Tim and Tom are the two mammals in Rovena’s garden, sounds fully satisfactory. Accordingly, in the AA^+^-proposal, the addition “*and there are no other mammals (than Tim and Tom) in her garden*” sounds superfluous. For *widow*, the A-proposal, “Xanthippe became a widow because Socrates died”, sounds fully satisfactory; accordingly, it would seem useless to add, as in the AA^+^-proposal, “*and Xanthippe was married to Socrates*”.

In general, the AA-proposal (unlike the AA^+^-proposal) seems to contain just enough to fully answer the question of why C obtains. Given (Explanation-Proposal), we conclude that AA (unlike AA^+^) contains just what is relevant to fully explain C. Yet, I submit, there is an ambiguity in the content of the AA-proposal; and it should be resolved before drawing any hasty conclusion. For simplicity, let us say that an AA-proposal for C is *successful* just when it meets the criterion considered: one would accept it as containing just enough for a fully satisfactory answer to why C obtains. Now, a successful AA-proposal allows for two interpretations: what we will call the *strict* (or minimal, or literal) content, including only the facts explicitly cited, AA; and the *broad* (or inclusive, or contextual) content, which also includes the facts which need to be *implicitly* implied or assumed for the AA-proposal to be successful. (More precisely, among those facts, some are only *trivially* needed: they obviously need to be implied or assumed for one to even be in a position to reasonably accept or reject the proposal, in particular to understand what is explicitly said, or recognize it as a meaningful explanatory proposal in the first place – *whether or not* one will then actually *accept* it as a satisfactory full explanation of C (e.g. facts about the reference or meaning of words used in the proposal, about grammatical rules, perhaps about what language is used by the speaker, her intention to offer an explanation, and so on). Other facts are *non-trivially* needed for the explanatory proposal’s success: they need to be implied or assumed, not (only) for one to even be in a position to reasonably accept or reject it, but (also) *specifically* to *accept* it as a satisfactory full explanation. Whenever I speak of the broad content as including the facts needed to be assumed or implied for the success of a proposal, it will always mean those *non-trivially* needed.^29^)

For *taste*, the broad/strict distinction may be elaborated as follows: the A-proposal may be interpreted *exhaustively* (as e.g. “I have two children” is typically interpreted, namely as implicitly implying that I have *no more* than two children) or *non-exhaustively* (e.g. “Do you have a car?” is typically *not* taken to imply *no more than one*).^30^ Most plausibly, the A-proposal is successful because we interpret it *exhaustively*: we are satisfied with “I’m having a pleasant tasting experience because I’m tasting chocolate” because we take it to imply that I am tasting nothing else than chocolate—i.e. in fact, we interpret it as the *AA*^+^-proposal. And it is the default interpretation in similar cases. If I told you that yesterday I was tasting the cake you made and found it delicious, you would probably be surprised (indeed disappointed or offended) if I later specified that, in fact, what I was tasting is a tiny bit of your cake together with a large quantity of another cake: that’s just *not* how you understood my initial claim – you took it as implying that I was tasting nothing else than your cake. Likewise, one would typically not take the A-proposal as just meaning “I’m having a pleasant tasting experience because I’m tasting *at least* chocolate”, without implicitly implying that I was tasting nothing else—thereby leaving it open whether I was simultaneously tasting fish or cheese or snails. Now, if the A-proposal *were* understood this way, as *not* implying that I was tasting nothing else, it would still make sense: it would be a different but meaningful explanatory proposal—but it would probably not be accepted as a satisfactory full explanation. Rather, it would plausibly lead to reactions of doubt, or to further questions—“So were you also tasting something else? What was it?”. And this is typically not a good sign that an explanation was fully satisfactory in the first place.

Similarly, for *squirrels*, the AA-proposal, “All mammals in Rovena’s garden are squirrels because Tim and Tom are squirrels”, is successful only because it is implicitly implied by the proposal, or anyway assumed in the background, that Tim and Tom are the only mammals in her garden. Otherwise, the AA-proposal would not sound like a fully satisfactory answer to why all mammals in her garden are squirrels. For *widow*, the A-proposal, “Xanthippe became a widow because Socrates died”, would not be successful if it were not implied or assumed that they were married. Rather, the proposal may lead to reactions of puzzlement—“But how is that relevant?”—or, at best, requests for a complement or confirmation—“Oh, so you mean Xanthippe was married to Socrates, right?”. (Just think of the analogous widow case with Socrates and Xanthippe replaced with complete strangers.) Such reactions are typically not good signs of a fully satisfactory answer. Likewise, for *obligation*, “I ought to φ because I promised to φ” is successful because we would typically interpret it as implying that I am able to φ and my promise was not given under duress. (You would probably be surprised if later on you learned that these further facts did not obtain–this is *not* what you implicitly got from the proposal.)

In sum, we first accept the AA-proposal for C as a fully satisfactory answer based on its *broad* content (including the non-explicit but indispensable facts SS); but then, from this success of the AA-proposal, (Explanation-Proposal) makes us conclude that its *strict* content is just what is relevant to fully explain C—*even* if the broad content, most plausibly AA^+^, is *distinct* from the strict content, AA *alone*. This discrepancy seems unjustified. Accordingly, I propose that we rather rely on a successful explanatory proposal’s *broad* content—whether all of it is explicit or not. The resulting alternative criterion may be formulated as follows:

(Explanation-Proposal*) *AA*^+^ contains just what is relevant to fully explain C *iff* there is a (strict or non-strict) subplurality AA⊆AA^+^ such that one would accept the AA-proposal as containing just enough for a fully satisfactory answer to the question of why C obtains, *and AA*^+^ *is the broad content of the AA-proposal*.

I already illustrated how, in our alleged counterexamples to GN, the broad content of a successful AA-proposal for C is most plausibly not the contingentist’s preferred candidate, AA, but the completed AA^+^. And I provided a first motivation for the above criterion itself–it avoids an intuitively unjustified discrepancy. To put it otherwise, since SS is at least clearly relevant in the sense that it *needs* to be implicitly implied or assumed for the explanatory proposal for C to be successful, it strongly suggests that SS is indeed part of what is relevant to fully explain C.

I will now further argue for (Explanation-Proposal*), and against (Explanation-Proposal), by presenting important problems for the latter, but not the former. In order not to beg the question, I will mostly rely on cases which are analogous to our alleged counterexamples to GN, but which are neutral in that the question of what fully explains or grounds what in those cases should not be a matter of dispute between contingentists and necessitarians.

Consider first *vixen*. Most plausibly, the plurality [*a* is a fox], [*a* is a female] fully grounds and fully explains [*a* is a vixen]—while neither [*a* is a fox] nor [*a* is a female] *alone* does. Yet, consider a situation where I am walking in a forest with my friend and I tell her: “Oh look there, it’s a fox!”. My friend, who happens to be a zoologist, kindly corrects me: “No, it’s a *vixen*, because it’s a female.” Now, in that context*,* where we know that the animal is a fox, her explanation of why it’s a vixen in terms of its being a female sounds fully satisfactory. Accordingly, “It’s a vixen because it’s a female *and it’s a fox*” might have sounded like containing too much–after all, *I* was the one who identified that animal as a fox in the first place! Given (Explanation-Proposal), we conclude that AA^+^ = [It’s a fox], [It’s a female] does *not* contain just what is relevant to fully explain C = [It’s a vixen], while [It’s a female] *alone* does. Assuming (GROUND-EXPLANATION), we further conclude that, after all, what intuitively looks like the obvious candidate, the plurality [It’s a fox], [It’s a female], cannot fully ground [It’s a vixen]–while [It’s a female] alone could. These conclusions sound highly implausible.

Likewise, consider *party*. Suppose that my friend and I organize a party in our flat and all of the 10 people we invited show up at the expected time, except one: Martin. So at the beginning of the party, my friend and I know and assume that there are 11 people in total in the flat. Two hours later, my friend wonders: “I’ve just quickly looked around and there are 12 people in here now–how come?” In fact, she did not notice that Martin had finally showed up. So I answer: “Oh yes, there are now 12 people because Martin is here!” My friend looks fully satisfied with my answer–it was just the explanation she needed. According to (Explanation-Proposal), [Martin is here] is just what is relevant to fully explain [There are 12 people here]–which sounds highly implausible.

Finally, take the more generic case of *conjunction* (particular instances may easily be imagined). It is (almost) uncontroversial that a conjunctive fact like C = [a ∧ s] is fully grounded and fully explained by the plurality of its conjuncts, AA^+^ = A, S–where A = [a], S = [s]. Yet, in a situation where it is tacitly assumed that s, the A-proposal for C, “s *and* a because a”, may well be successful, and (Explanation-Proposal) would lead us to the highly implausible conclusion that [a] is enough to fully explain [a ∧ s].

Such cases seem to clearly speak against (Explanation-Proposal). By contrast, they are unproblematic for (Explanation-Proposal*). Most plausibly, in *vixen*, where it is tacitly assumed that it’s a fox, the plurality AA^+^ = [It’s a female], [It’s a fox] is the *broad* content of the successful A-proposal–while A = [It’s a female] alone is not the broad content of this or any other successful explanatory proposal. Given (Explanation-Proposal*), we correctly conclude that the plurality AA^+^ = [It’s a fox], [It’s a female] contains just enough to fully explain [It’s a vixen]–while A = [It’s a female] alone does not. By similar reasoning, (Explanation-Proposal*) correctly rules out [Martin is here] alone as fully explaining [There are 12 people here], and [a] alone as fully explaining [a ∧ s].

Beyond such plausible counterexamples, (Explanation-Proposal) is in clear tension with two very plausible, indeed widely shared, ideas about grounding. First, grounding is an *objective*, mind-independent relation; and so is the corresponding sort of metaphysical explanation (whether the former *is* or only *backs* the latter).^31^ In particular, whether or not AA fully grounds or explains C should not depend on what is implicitly implied or assumed in a particular context. Yet, for instance, if A = [It’s a female] and C = [It’s a vixen], then whether the A-proposal for C would be successful plausibly depends on whether we consider a context where S = [It’s a fox] is tacitly assumed to obtain. Given (Explanation-Proposal), this context-dependence is transmitted to whether [It’s a female] contains just enough to fully explain [It’s a vixen]—and, therefore, to whether the former meets the necessary condition in (GROUND-EXPLANATION) to fully ground the latter. Similarly for our alleged counterexamples to GN: whether or not one would be fully satisfied with “All mammals in Rovena’s garden are squirrels because Tim and Tom are squirrels” depends on whether or not it is implied or assumed that Tim and Tom are the only mammals in her garden; given (Explanation-Proposal), this dependence is transmitted to whether the relevant relation of full metaphysical explanation holds.

In general, whether a *verbal* explanation is successful is notoriously context-dependent; and (Explanation-Proposal) inherits this dependence since it simply takes the facts explicitly cited in a successful verbal explanation to provide a full *metaphysical* explanation. By contrast, (Explanation-Proposal*) relies on the *broad* content of successful verbal explanations, which remains more stable across contexts – what does vary is rather the division between the explicit and implicit parts *within* that broad content. For instance, on (Explanation-Proposal*), whether A = [It’s a female] fully explains C = [It’s a vixen] does *not* depend on whether we are in a context where S = [It’s a fox] is assumed to obtain or not: *either way*, the most probable successful proposal (the A-proposal in one case, the AA^+^-proposal in the other) has AA^+^, not A, as its broad content.

Second, and relatedly, grounding and the corresponding metaphysical explanation are *systematic* relations: whether they hold should not depend on what specific examples we choose from a category of relevantly analogous cases–if [Greg’s shirt is scarlet] fully explains/grounds [Greg’s shirt is red], then [Alex’s shirt is scarlet] should also fully explain/ground [Alex’s shirt is red].^32^ Now, in *widow*, for instance, (Explanation-Proposal) led us to conclude that A = [Socrates died] *alone* fully explains C = [Xanthippe became a widow]–since we know and tacitly assume that S = [Socrates and Xanthippe were married] obtains, we would plausibly be fully satisfied with the A-proposal. However, if Jane and John are complete strangers, we would most plausibly *not* be fully satisfied with “Jane became a widow because John died”. Given (Explanation-Proposal), we conclude that [John died] does *not* fully explain [Jane became a widow]. Yet, the two cases are perfectly analogous in all relevant respects – systematicity is lost. By contrast, it should be clear how (Explanation-Proposal*), relying on *broad* content, would treat both cases analogously.

Before I conclude, let me address a potential rejoinder. Given the above, the contingentist may accept that (Explanation-Proposal) goes too far in *always* relying on strict content; but, he may add, so does my proposed (Explanation-Proposal*), in *always* relying on broad content–including the non-explicit SS. Rather, we should go for a “mixed” criterion, (Explanation-Proposal**): for a successful proposal for C, just what is relevant to fully explain C is the *broad* content of the proposal *in cases of type 1*, and its *strict* content *in cases of type 2.* Type 1 at least includes all the plausible neutral counterexamples to (Explanation-Proposal)—e.g. *vixen*, *conjunction* –, and type 2 includes at least (some of) our alleged counterexamples to GN—e.g. *widow*, *squirrels*. In that way, (Explanation-Proposal**) would (very conveniently) both avoid the plausible neutral counterexamples to (Explanation-Proposal), and “save” (some of) the alleged counterexamples to GN.

However, for this suggestion to be more than a purely ad hoc move to save contingentism, much clarification and argument would be needed. First, type 1 or 2 cannot just be *anything* which at least includes the particular cases that the contingentist needs it to include. Rather, each type in general should first be given a clear and independent characterization. Second, on that basis, it should be shown that each type indeed includes the cases that it is supposed to. Third, crucially, the contingentist would owe us a clear and independent reason *why* we should rely on the broad content of successful proposals in cases of type 1, but on strict content in cases of type 2: that is, why SS—which, in *all* cases considered, always contains facts which, if not explicit in the explanatory proposal, are still relevant in that they need to be implied or assumed for it to be successful—should clearly be taken into account as explanatorily relevant in cases of type 1, but just as clearly be ignored in cases of type 2. The suggestion under consideration simply cannot take off the ground until these questions are satisfactorily answered. Moreover, even on the (*prima facie* improbable) assumption that they could be, the mixed (Explanation-Proposal**) might “save” (some) counterexamples to GN, and avoid plausible neutral counterexamples to (Explanation-Proposal), but since it would still partly rely on *strict* content, it would presumably still face the *further*, general problem of undermining the objectivity and systematicity of grounding and metaphysical explanation.

I conclude that (Explanation-Proposal) should be rejected, and replaced with (Explanation-Proposal*). On this amended criterion, in our alleged counterexamples to GN, AA (unlike AA^+^) fails to contain just what is relevant to fully explain C, because it fails to be the broad content of a successful explanatory proposal for C. Consequently, AA (unlike AA^+^) fails to meet necessary condition (GROUND-EXPLANATION) to fully ground C—the alleged counterexample is not genuine. If moreover C is fully grounded and AA^+^ is the only serious rival, we should indeed conclude that AA^+^ fully grounds C.

### Generative relevance: creation metaphors and background assumptions

My second argument is distinct, though analogous in important respects (so that it should remain clear even with some details left implicit). Beyond metaphysical explanation, grounding is also widely thought to be closely connected to metaphysical *generation*—or determination, or building.^33^ Indeed, some (separatists) take grounding to just *be* (primarily) a form of generation—generation_G_. For them in particular, it should be most natural to think that a ground is wholly generatively relevant to what it grounds, and that a *full* ground, moreover, contains *all* of what is relevant for it to *fully* generate what it grounds—i.e. it contains no more and no less than what it needs to do so.

**(GROUND-GENERATION)** If AA fully grounds C, then AA contains just what is relevant to fully generate C.

(GROUND-GENERATION) should be most appealing to separatists, but still acceptable to some unionists. Importantly, like (GROUND-EXPLANATION), (GROUND-GENERATION) is acceptable to both contingentists and necessitarians.

A common way to assess whether AA, a candidate full ground for C, meets (GROUND-GENERATION) is to appeal to a creation metaphor (see Leuenberger, 2008, 2014a; Schaffer, 2009: 351; Skiles, 2015; Cameron, 2018). Calling the “AA-decree” the divine decree citing the facts in AA – “Let AA obtain”, or equivalently, “Let it be the case that *p*, *q*, …”, with AA = [p], [q], …–, the idea is the following:

**(Generation-Decree)** AA contains just what is relevant to generate C *iff* one would accept God’s uttering the AA-decree as being just enough to fully generate C.

Ultimately, I will argue against (Generation-Decree), and defend a better alternative. But again, it will be instructive to first assume it and see how it plays in favour of the contingentist’s position.

For simplicity, instead of a restricted generalization ([∀x(Fx → Gx)]), let us first consider an *unrestricted generalization*: C = [∀xFx]. What would God need to decree in order to generate C? For AA = [Fa], [Fb], … – where *a*, *b*, … are all the things that exist –, the AA-decree is “Let *a*, *b*, … be F”. For AA^+^ = AA, S–where S = [*a*, *b*,… are all the things that exist] –, the AA^+^-decree is “Let *a*, *b*, …be F *and let nothing else exist*”. AA seems to satisfy (Generation-Decree); accordingly, AA^+^ does not: the AA^+^-decree seems to contain more than needed. For intuitively, once God has decreed that *a*, *b*, … be F, She can just *stop*. No further command is needed to fully generate C–since everything is F, C *already* obtains! Commanding, *in addition*, that *a*, *b*,… be the only things that exist would thus be superfluous (see Skiles, 2015, §4.1). Similarly, in our *restricted* generalization, *squirrels*, it seems that God’s commanding that Tim and Tom (the two mammals in Rovena’s garden) be squirrels would be just enough to fully generate the fact that all mammals in Rovena’s garden are squirrels. For *widow*, it seems that God’s commanding that Socrates cease to exist would be just enough to make Xanthippe a widow.

Although I appreciate the intuitive appeal of this line of thought, it would be too hasty to conclude—as (Generation-Decree) would require–that AA (unlike AA^+^) contains just what is relevant to fully generate C—or so I will now argue. In the context of a creation metaphor, we are assuming that certain facts obtain as part of the tacit background for God’s intervention, including facts without which the AA-decree would not be successful—i.e. we would not accept that God’s formulating it is just enough to fully generate C. In *unrestricted generalization*, we tacitly assume that, before uttering the AA-decree—“Let *a*, *b*,… be F” –, God created *a*, *b*,… and nothing else. We also tacitly assume that God is the *only* creator—e.g. that no other being may, just when God utters the AA-decree, command *further* (perhaps non-F) things to exist. Moreover, if the creation metaphor is meant to concern whether God’s uttering the AA-decree at a *previous* time made C = [Everything is F] obtain *now*, then the AA-decree will probably not be successful unless we further assume that–as the contingentist emphasizes–God *stopped* after uttering Her decree, indeed that She stopped *once and for all*–excluding that She might have stopped just for a whisky break and then come back to create further (perhaps non-F) things. In any case, for us to accept that God’s uttering the [Let *a*, *b*,… be F]-decree is just enough to fully generate [Everything is F], we most plausibly need to tacitly assume facts which together amount to the totality fact S = [*a*, *b*,… are all that exists]. Likewise, we may accept that God’s commanding that Tim and Tom be squirrels is just enough to make all mammals in Rovena’s garden squirrels, but only if we imagine a pre-intervention situation where Tim and Tom are the only two mammals in her garden. And we may accept that God’s commanding that A = [Socrates dies] obtain would be just enough to generate C = [Xanthippe becomes a widow], but only because we imagine a situation where S = [Xanthippe and Socrates are married] obtains.

Thus, beyond the facts which God explicitly commands to obtain, AA, there are further facts SS which are clearly relevant in that, without assuming that they obtain, the AA-decree for C would not be successful. Should we exclude them from what is relevant to fully generate C, as (Generation-Decree) requires? Consider a case which is analogous but should not be a matter of dispute between necessitarians and contingentists: *round* + *blue*. Suppose that S = [*a* is round] is tacitly assumed to obtain as part of a creation metaphor’s background. And you ask: *now*, what would be a divine decree such that God’s uttering it would be just enough to fully generate C = [*a* is round and blue]? It seems that the [*a* is blue]-decree, namely “Let *a* be blue”, would do. Applying (Generation-Decree), we should conclude that A = [*a* is blue] is enough to fully generates C–which is implausible. In the same tacit pre-intervention background, one may further argue that God’s uttering the AA^+^-decree–where AA^+^ = [*a* is round], [*a* is blue]–would be too much. Given (Generation-Decree), we should conclude that AA^+^ fails to contain just what is relevant to fully generate [*a* is round and blue]—which is again implausible –, so that the former fails to meet necessary condition (GROUND-GENERATION) to fully ground the latter—although it looks like the *obvious* candidate.

This seems to clearly speak against (Generation-Decree). And it suggests how (Generation-Decree) may undermine the widely shared idea that grounding and the corresponding metaphysical generation are *objective* relations—they should not depend on what is tacitly assumed in the context of a creation metaphor. For instance, whether the [*a* is blue]-decree is successful most plausibly depends on whether [*a* is round] is part of the tacit pre-intervention background; given (Generation-Decree), this dependence is transmitted to whether [*a* is blue] is just enough to fully generate [*a* is round and blue]. Likewise, for *unrestricted generalization*, the success of the [Let *a*, *b*,… be F]-decree depends on whether it is assumed that [*a*, *b*,… are all that exists] obtains; given (Generation-Decree), the dependence is transmitted to whether the relevant relation of full generation holds.

Relatedly, (Generation-Decree) threatens the very plausible idea that grounding and the corresponding metaphysical generation should be *systematic*. For instance, it is (almost) uncontroversial that a conjunctive fact is fully grounded and fully generated by the plurality of its conjuncts; and this should not depend on the *particular* conjunctive fact considered. Yet, as *round* + *blue* suggested, such systematicity may be lost with (Generation-Decree). Likewise, it is easy to see how, given (Generation-Decree), two perfectly analogous “widow facts” may be fully generated and fully grounded in different ways depending on whether the specific widow is Xanthippe or Jane, a complete stranger who was married to John, another complete stranger (since we know and would naturally assume that Xanthippe and Socrates were married as part of the pre-intervention background, while we would have no reason to assume that Jane and John were married).

(Generation-Decree) has such problematic consequences for essentially the same reason as (Explanation-Proposal): (Generation-Decree) only relies on the facts explicitly commanded by God in a successful AA-decree—the decree’s *strict* content–, leaving out the facts which (non-trivially^34^) need to be tacitly assumed for the decree to be successful—i.e. the implicit part its *broad* content. Rather, we should rely on its *entire* broad content:

(Generation-Decree*) *AA*^+^ contains just what is relevant to generate C *iff* for some (strict or non-strict) plurality AA⊆AA^+^, one would accept God’s uttering the AA-decree as being just enough to fully generate C, *and AA*^+^ *is the broad content of the AA-decree*.

This criterion avoids the abovementioned problems with (Generation-Decree). We most plausibly need to assume that S = [*a* is round] obtains in order to accept that God’s uttering the [*a* is blue]-decree is just enough to fully generate C = [*a* is round and blue], so that A = [*a* is blue] is not the broad content of the A-decree–or of any plausibly successful divine decree for C. Therefore, (Generation-Decree*) correctly leads us to conclude that A is *not* just what is relevant to fully generate C. Likewise, it should be clear how (Generation-Decree*) also leads us to the correct conclusion that the plurality AA^+^ = [*a* is round], [*a* is blue] does contain just what is relevant to fully generate C = [*a* is round and blue], since AA^+^ is most plausibly the broad content of a successful decree for C.

Moreover, it should be clear how (Generation-Decree*), relying on broad content, can better preserve the *objectivity* and *systematicity* of grounding and metaphysical generation: what may vary from one tacit pre-intervention background to another, or from one specific example to another within a family of analogous cases, is the division between the explicit and implicit part *within* the broad content of a successful divine decree – rather than the broad content itself.^35^

I conclude that (Generation-Decree) should be rejected, and replaced with (Generation-Decree*). On the amended criterion, in our alleged counterexamples to GN, AA (unlike AA^+^) fails to contain just what is relevant to fully generate C, because it fails to be the broad content of a successful decree for C; consequently, AA (unlike AA^+^) fails to meet necessary condition (GROUND-GENERATION) to fully ground C – the alleged counterexample is not genuine. If furthermore C is fully grounded and AA^+^ is AA’s only serious rival, we should indeed conclude that AA^+^ fully grounds C.^36^

### Overkill: grounding regularity lost

My third, indirect argument may be equally appealing to unionists and separatists. The intended target of the contingentist’s alleged counterexamples is the strong claim that all full grounds *metaphysically necessitate* what they ground (GN). Yet, it turns out that, if indeed genuine counterexamples – i.e. if AA fully grounds C –, they falsify modally weaker views which even contingentists may want to preserve.

To illustrate, consider first grounding *nomic* necessitarianism (GNN): if AA fully ground C, then ☐_N_(AA → C)—where ☐_N_ is the nomic necessity operator. GNN is weaker than GN, assuming the common view that nomic necessity is weaker than metaphysical necessity.^37^ Yet, if genuine, our four illustrative alleged counterexamples to GN would also falsify GNN. The key point to notice is that, to imagine the contingentist’s scenarios, where AA obtain with some blocker BB and C does not (Socrates died, Socrates and Xanthippe were never married, Xanthippe did not become widow), you typically do not need to imagine remote metaphysically possible worlds – with alien kinds or strange laws of nature. You just need to consider worlds which are quite similar to ours, and clearly *nomically* possible. Thus, if we accept with the contingentist that AA fully grounds C, we get counterexamples not only to GN but the weaker GNN – i.e. ◇_N_(AA∧BB∧¬C). This holds not only for alleged counterexamples to GN of the type considered here, but of *any* type, provided that they involve a nomically possible scenario – and most counterexamples presented in the literature clearly do.^38^

Indeed, many alleged counterexamples to GN (and GNN), if successful, would falsify the still weaker claim that grounding relations involve mere *regularities*–corresponding universal generalizations, even without any modal force. A natural way to formulate that claim, *grounding regularity* (GR), is with *types* of facts – calling e.g. “A-like” facts all actual facts of the same type as A (i.e. all actual facts which are the same as A *except* that they need not obtain at the same time and place). GR says that, if AA fully grounds C, then all actual AA-like pluralities of facts co-obtain with (i.e. obtain at the same time and place as) a C-like fact. Now recall *taste*: A = [I’m tasting chocolate] fully grounds C = [I’m having a pleasant tasting experience], but there is a possible scenario where A obtains together with B = [I’m tasting fish] and C does not. This scenario is not only metaphysically (indeed nomically) possible: there is another *actual* situation (which happened yesterday, say) where I was tasting both chocolate and fish at once and my tasting experience was terrible. Thus, an actual A-like fact fails to co-obtain with a C-like fact—the corresponding regularity is lost. A similar argument would work for many other cases of the type considered here, including *obligation* and *squirrels*—indeed, for *any* type of alleged counterexample to GN involving a scenario violating a corresponding regularity.

Clearly, in all the relevant cases, GNN or even GR is lost assuming that AA indeed fully grounds C; by contrast, with AA^+^ as a full ground, GN is preserved, and so are, *a fortiori*, GNN and GR. The claim that, for instance, (some) *causal* relations do not even correspond to universal generalizations may have some plausibility–e.g. because (some) causal laws are mere approximations, or some instances of causation are irreducibly indeterministic. But it sounds less plausible that grounding involves a similar form of approximation or indeterminacy: if an A-like fact fully grounds a C-like fact on some occasion, but on other *actual* occasions A-like facts sometimes do and sometimes do not come with a C-like fact, then grounding would look somehow fuzzy and capricious–arguably not what one would expect from a robust, *metaphysical* relation.^39^ Unlike GN, the much more moderate idea that full grounding involves at least (non-accidental) regularity sounds acceptable to, and may have some plausibility for, necessitarians and contingentists alike–as indeed some discussions of grounding on both sides suggest.^40^ Anyone wanting to preserve that moderate idea has at least one good reason not to take AA to fully ground C in the relevant cases–and to go for AA^+^ instead.^41^,^42^

## Conclusion

I have addressed a typical family of alleged counterexamples to GN for which the debate essentially reduces to a choice between two main plausible candidate full grounds for a fact C: AA and AA^+^. I argued that, unlike AA^+^, AA should not be taken to fully ground C–not because AA^+^ necessitates C and AA does not, but for three independent reasons. Unlike AA^+^, AA (i) fails to fully explain C, (ii) fails to fully generate C, and (iii) fails, if taken as a full ground, to preserve even grounding regularity. Relying on different bases, the three arguments may convince different readers. The first may be most appealing to unionists, the second to separatists–and assuming that most readers are either unionists or separatists, most may find some appeal in at least one. My third argument may be appealing to unionist and separatists alike–or to readers who are on neither side.

My defence, even if convincing, may not establish GN in all generality (excluding *any possible* counterexample), since I focused on a specific type of counterexample. However, that type plays a particularly important role in the debate about GN; and the potential applicability of the completer strategy defended is wide, likely to go beyond that category.^43^ Thus, if sound, the proposed defence still brings significant support to GN.

## Footnotes

1 On grounding, see e.g. Fine (2001, 2012b), Correia (2005), Schaffer (2009), Rosen (2010), Correia & Schnieder (2012); for some critical views, see e.g. Sider (2012), Daly (2012), Wilson (2014), Koslicki (2015), Hofweber (2016), Thompson (2018).

2 My discussion of necessitarianism is restricted to *metaphysical* grounding, understood as a (sufficiently) unified phenomenon. Some argue that there are distinct varieties of grounding beyond metaphysical grounding (e.g. natural or normative grounding–see Fine (2012b)), or that metaphysical grounding itself is in fact a variety of distinct phenomena (see e.g. Wilson 2014; Richardson 2021). Whether and how my arguments may also apply or adapt to (some of) these varieties is an interesting question, but not one I can hope to address here.

3 Necessitarian views include Correia (2005), DeRosset (2010, 2013a, 2013b), Rosen (2010), Audi (2012a, 2012b), Fine (2012b, 2015), Trogdon (2013a), Dasgupta (2014), Wilsch (2015); defences of contingentism (i.e. the negation of GN) include Dancy (2004 (ch.3)), Schnieder (2006a, 2006b), Schaffer (2010), Chudnoff (2011, ms), Leuenberger (2014a), Skiles (2015). Richardson (2021) argues for a pluralist view–there are two kinds of grounding, only one involving necessitation.

4 Following Skiles (2015), contingentism here is the negation of GN (i.e. the view that at least some full grounds are non-necessitating).

5 I borrow the enabler/completer terminology from Chudnoff (ms).

6 See e.g. Rosen 2010; Audi 2012b. Others formulate grounding as a sentential connective—see Fine 2001, 2012b; Correia 2010. Such fine-grained distinctions will not be crucial here.

7 For a similar ‘blocker’ objection against *causal* necessitarianism, see Russell (1912: 7), Schrenk (2010), Mumford & Anjum (2011, 2014); for replies, see Lowe (2012), Mumford & Anjum (2014: 113–115,) Marmodoro (2015), Hirèche (2021). Some relevant (dis)analogies will be mentioned (see footnotes 36, 42 in particular).

8 The necessitarian might claim that the relevant notion is not necessitation *tout court*, but some qualified notion allowing for exceptions–e.g. necessitation *ceteris absentibus* (Leuenberger 2008) or *in the absence of blockers* (Bird 2005). Alternatively, she might argue that necessitation is *non-monotonic* (like Lowe’s (1995) “if–then” relation, or Fine’s (2012c: 235–6) “exact entailment”). Lowe (2012) pursues a similar strategy against Mumford and Anjum’s (2011) blocker objection to *causal* necessitarianism (for discussion, see Hirèche 2021: §3.3).

9 Perhaps with further facts about my tasting preferences.

10 In particular, it presupposes that the co-obtaining of an alleged full ground A and blocker B is correctly formalized as A∧B. Yet, one may argue that the “composition” of A and B is not “classical”: it amounts to a “holistic” fact H, which does not include the individual A or B. Accordingly, the contingentist’s scenario is correctly formalised, *not* as ◇(A∧B∧¬C), but as ◇(H∧¬C)—the counterexample is lost. This strategy was used by J. Heil and O. Massin to defend *causal* necessitarianism against analogous blockers (Mumford & Anjum 2014: 113–115); see also Hirèche (2021: §4). It may work for specific grounding cases (e.g. perhaps *taste*); for the rest (e.g. *widow*), it looks hopeless.

11 On totality facts, see Russell 1918: 207; Armstrong 1997: ch. 13; Chalmers & Jackson 2001; Heil 2003: 70–71; Rosen 2010: §8; Leuenberger 2014b; Fine 2015: §7; Krämer forthcoming. One worry is that totality facts include *negative* facts, which seem ill-suited to ground anything (see Mumford & Anjum (2011a: 66; ch. 6) for the *causal* analogue; Mellor (1995: 131–5) for an opposite view). Another worry concerns *relevance* (Skiles 2015: §4.2): suppose totality fact T = [∀x(Fx → (x = a ∨ x = b ∨…))] partially grounds C = [∀x(Fx → Gx)]. T is plausibly grounded in a huge collection including facts specifying e.g. which things are *not* F. Given grounding transitivity, C is partly grounded in facts plausibly irrelevant to it. Perhaps both worries are addressed by replacing T with the fact, T_F_(*a*, *b*,…), that *a*, *b*,*…* are *exactly* the F’s (Armstrong 1997: 199–200; Fine 2012b). T_F_(*a*, *b*,…) may necessarily co-obtain with [Fa], [Fb], …, *and T* together, but still be *distinct* and not reduce to them. Skiles (2015: §4.2) worries about the ontological cost of postulating facts like T_F_(*a*, *b*,…) as basic. Anyway, such potential costs of T or T_F_(*a*, *b*,…) partially grounding C may be acceptable if, as I will argue, C could *not* be fully grounded otherwise.

12 Focusing on cases where C most plausibly does have a full ground will not restrict the scope of my defence: GN is trivially compatible with any case where C has no full ground (see Trogdon & Witmer 2021).

13 I am not assuming a strong principle like Minimality: If AA fully grounds C, then any AA^+^ strictly containing AA does not fully ground C (Audi 2012b: 699). Minimality faces counterexamples involving e.g. conjunctions with one conjunct fully grounding the other (Dixon 2016), or infinite disjunctions of infinite conjunctions (Fine 2012b, 57); Werner (2020) argues that they are addressed if Minimality is restricted to immediate grounding. Even if Minimality does not hold in all cases, the motivations behind it–concerning relevance (see e.g. Michels (2018, 806–8))—still plausibly apply to many, including those considered here.

14 See e.g. Rosen (2010: §8), Trogdon (2013a), Fine (2015: §7).

15 See e.g. Dancy (2004, ch. 3), Leuenberger (2014a), Skiles (2015), Richardson (2021), Chudnoff (2011, ms).

16 Existing general defences of GN rely on e.g. explanation (DeRosset 2010), essence (Audi (2012b), Correia & Skiles (2019), Dasgupta (2014), Fine (2012b), O’Conaill (2018), Trogdon (2013a)), metaphysical laws (Wilsch 2015), grounding internality (Lovett 2019). For some responses, see e.g. Skiles (2015), Richardson (2021).

17 See Trogdon (2013a: 478).

18 E.g. *Physicalism*: Some physical facts AA fully ground phenomenal fact C, but there could have been an alien property blocking phenomenal experience (see Leuenberger 2014a: II.i) The present discussion may be relevant to this case, for which the completion strategy looks promising. However, here a decision would first have to be made among more than one reasonable candidate completers–e.g. a totality fact (see Leuenberger 2014a: 158–9) or a “no-blocker” fact (see Richardson 2021: §3.1). Moreover, other strategies are plausible—e.g. O’Conaill (2018) addresses this case relying on grounding’s links with essence. Similar remarks apply to Leuenberger’s (2014a: II.ii) other counterexample, *categoricalism* (categorical facts fully ground dispositional facts, but there are possible blockers). Another type of case to mention is *material composition*: C, the fact that material object *o* exists, is fully grounded in facts AA about the arrangement of *o*’s parts, the *p*’s. However – after the *p*’s “scatter” away and later come back to their initial arrangement –, AA could obtain without C (Skiles 2015: §3). Some of my discussion may apply to this case, though it is not typical of those I focus on. It is not (obviously) understood as involving a blocker; and although the completer strategy may apply here, there would again be more than one reasonable candidate—e.g. the fact that the *p*’s compose *o*, or facts about *o*’s origin (see DeRosset 2013b: §5; Skiles 2015: §3.1).

19 E.g. Rosen (2010: 2), Fine (2012a, 2), Audi (2012b: 699), Dasgupta (2014, 4), Litland (2016: 534), Michels (2018: 806).

20 Including Fine (2012b: 37), Rosen (2010), Raven (2012), Litland (2013), Dasgupta (2014).

21 Including Audi (2012b), Koslicki (2012), Schaffer (2012), Trogdon (2013b), Maurin (2019).

22 On the unionists/separatists divide, see e.g. Raven (2015), Bliss & Trogdon (2021), Skiles & Trogdon (2021).

23 See e.g. Fine (2001: 15), Fine (2012b: 37), Rosen (2010), Raven (2012), Litland (2013), Dasgupta (2014).

24 See e.g. Rodriguez-Pereyra (2005), Audi (2012b: 687–8), Schaffer (2012, 2016: 58–59), A. Wilson (2018: 728–9.)

25 Some have argued against the mind-independence of metaphysical explanation, or indeed of grounding itself (see e.g. Thomson (2016, 2018), Maurin (2019)).

26 See e.g. Correia (2010: 263), Schnieder (2011: 450), Audi (2012b: 693, 699), Fine (2012a: 2), Fine (2012b: 56), Raven (2013: 198), Dasgupta (2014: 4). But this is disputed–see e.g. Wilson (2014), Krämer & Roski (2017), Lange (2019).

27 See e.g. Dixon (2016: 375).

28 In particular, they may argue that some AA might fully ground some C without containing all what is relevant to fully explain C: C might instead be fully explained by a plurality AA^+^, containing exactly AA and further facts SS (see Skiles 2015: §5.4.). More precisely, what is relevant to fully explain C can be divided into (i) the full ground “proper”, AA; and (ii) some external conditions SS “enabling” AA to fully ground C (see Chudnoff 2011; Chudnoff, ms; Dancy 2004: ch. 3). However, following Trogdon (2013a: 483), such a distinction between (i) and (ii) might be disputed on the grounds that it is likely to be largely pragmatic or perspectival (rather than metaphysical), just like the analogous distinction between cause and surrounding conditions probably is (see e.g. Mumford & Anjum 2011: 32; Heil 2012: 126–130)—which may be problematic, in particular, if grounding itself is supposed to be a robust, metaphysical relation. (For more favourable takes on the ground/enabler distinction, see Moran 2018; Cohen 2020; Baron-Schmitt 2021).

29 Excluding the facts only *trivially* needed from the broad content is meant to avoid an important objection to the alternative criterion that I will soon propose, (Explanation-Proposal*), which takes what fully explains C to be the *broad* content of a successful proposal for C. If the broad content simply included *all* the facts needed to be implied or assumed for success, then facts which are plausibly irrelevant to explaining C (e.g. facts about the meaning of words in the proposal) would still have been part of what fully explains C according to the criterion (thanks to an anonymous reviewer for raising this issue). By restricting the broad content to only the facts non-trivially needed for success, the problem is hopefully addressed (at least to a large extent). Note that this type of restriction of the broad content may be replaced (or supplemented) with an alternative one, for which a trivial/non-trivial distinction is not required: the broad content of a successful AA-proposal is AA^+^ = AA, SS (with SS possibly empty), where SS are the facts such that, if they were not implied or assumed, then the AA-proposal would not be successful *but the AA*^+^*-proposal (explicitly stating SS) would be successful*. This also looks like an efficient way to exclude explanatorily irrelevant facts, since a proposal *explicitly* stating them would probably not be successful.

30 On exhaustive interpretation, see e.g. Schulz & Van Rooij (2006).

31 The mind-independence of grounding and metaphysical explanation are disputed, however (see e.g. Thompson 2016, 2018; Maurin 2019).

32 See Dasgupta (2014: 570), Audi (2012b: 693), Schaffer (2016: 57), Tugby (2022: 2604).

33 See e.g. Fine (2012b (§1)), Skiles (2015 (§1)), Schaffer (2016: 50), Bennett (2017).

34 I am assuming the same sort of trivial/non-trivial distinction as for explanatory proposals (§3.1). That is, the implicit part of the broad content of the AA-decree does *not* include the facts only *trivially* needed for the decree’s success, namely those one needs to assume to even understand what the decree explicitly says in the first place, and more generally to even be in a position to reasonably assess whether God’s uttering the AA-decree is just enough to fully generate C (e.g. facts about the meaning of words used in the decree, about what C amounts to, or what it means that God’s uttering the decree is just enough to fully generate C); rather, it only includes the facts *non-trivially* needed for success, namely those one needs to assume *specifically* to *accept* that God’s uttering the decree would be just enough to fully generate C (e.g. [Xanthippe was married to Socrates]). Again, an alternative characterization doing without the trivial/non-trivial distinction might be preferable overall: the implicit part of the broad content just includes the facts SS (if any) such that, if they were not assumed to obtain, then the AA-decree would not be successful–*but the AA*^+^*-decree (with AA*^+^ = *AA, SS), stating SS explicitly, would be successful*.

35 At this point, the contingentist might again suggest a mixed criterion, (Generation-Decree**)—a “generative” version of (Explanation-Proposal**) which would be equally unpromising, for analogous reasons (see §3.1).

36 One may worry that my arguments (§§3.1–3.2) might commit me to *causal* necessitarianism (CN)—a view rejected by many, including grounding necessitarians (e.g. Rosen 2010: 118; Fine 2012b, §1)—because they mainly rely on grounding being a relation of explanation and generation, like causation (Schaffer 2016: 58–59; A. Wilson 2018: 728–9). So why should those same features not also serve to defend CN? (See Leuenberger 2014a:152; Skiles 2015: §4.1, §5.4, §6). My arguments might have been adapted to directly defend CN had I argued that, *in general*, what fully explains/generates must also necessitate—arguably, full causes should also be fully explanatory/generative, so they should also be necessitating. Yet, as I emphasized, my defence of the completion strategy was specific: I relied on explanation/generation to argue, *not* for GN in general, but specifically for the claim that, in an important type of objections to GN, the alleged full ground AA cannot be one without being completed with SS. At most, my arguments might adapt to defend CN *against the analogous type of objections* (see footnote 7). But nothing ensures that they would; for instance, in causal cases, it is often difficult to find a clear, plausible candidate completer in the first place—one excluding the alleged blockers, while not making necessitation purely vacuous (see e.g. Martin 1994: 5–6; Mumford & Anjum 2011). Moreover, while this type of objections is typical and arguably the most serious threat to GN, CN faces *other* equally serious threats that have no (equally plausible) grounding analogue (e.g. irreducibly indeterministic causation).

37 See e.g. Lewis (1973), Armstrong (1983), Fine (2005), Lange (2009), Mumford & Anjum (2011); for others, nomic necessity is as strong as metaphysical necessity–e.g. Ellis (2001), Bird (2007).

38 Two plausible exceptions are Leuenberger’s (2014a) cases, *physicalism* and *categoricalism*; however, my previous arguments (§§3.1–3.2) may be more relevant to them (see footnote 18).

39 Some might reject such considerations based on the view that there can be metaphysical indeterminacy (see e.g. Barnes 2014).

40 See e.g. Rosen (2010: 131), Audi (2012b: 693), Dasgupta (2014: 570), Schaffer (2016: 57), A. Wilson (2018: 729), Tugby (2022: 2604).

41 The contingentist willing to preserve GR may go for AA^+^ instead of AA in all cases where going for AA would violate a regularity, but still go for AA in other cases–which would suffice as counterexamples to GN. Yet, to avoid being completely ad hoc, this divide would need an *independent* justification–and both types of cases look analogous in all relevant respects (e.g. explanatory/generative relevance).

42 One may worry (again–see footnote 36) that a causal analogue of this argument commits me to (the plausibility of) *causal* necessitarianism. But even on the (disputable) assumptions that this commitment would be a significant drawback, and my argument can indeed be adapted to the causal case, it would have less force: arguably, the idea of perfect causal regularities is more disputable–indeed disputed–than its grounding analogue; in particular, it faces serious objections which have no plausible grounding analogue (e.g. irreducibly indeterministic causation).

43 See in particular the end of §2, and footnotes 18, 38.
